# The Flanged Hook Technique for Sutureless Refixation of Late Intraocular Lens-Capsular Bag Complex Dislocation: A Case Report

**DOI:** 10.7759/cureus.111278

**Published:** 2026-06-22

**Authors:** Hiroshi Shimizu

**Affiliations:** 1 Ophthalmology, Tawayama Shimizu Eye Clinic, Matsue, JPN

**Keywords:** capsular bag complex, flanged intrascleral fixation, intraocular lens dislocation, polypropylene iris hook, sutureless fixation

## Abstract

Late intraocular lens (IOL)-capsular bag complex dislocation is an important delayed complication after cataract surgery. This single-patient case report describes a sutureless anterior-segment approach using an unmodified commercially available polypropylene iris hook, which is referred to in this report as the Flanged Hook Technique. A 75-year-old man underwent uneventful cataract surgery, followed by neodymium: YAG posterior capsulotomy 4 months later. Approximately two years after cataract surgery, he developed symptomatic decentration of the IOL-capsular bag complex, and surgical repositioning was planned. After limited anterior vitrectomy through corneal side-port incisions, the straight end of a polypropylene iris hook was inserted into the lumen of a transconjunctival, 30-gauge, thin-walled needle, engaged with the anterior capsulorhexis margin, externalized by withdrawing the needle, thermally flanged with low-temperature cautery, and buried intrasclerally. The existing IOL-capsular bag complex was preserved using this sutureless anterior approach, without IOL explantation. During the limited three-month follow-up period, centration of the IOL-capsular bag complex was maintained without flange exposure, hook disengagement, recurrent decentration, or other notable ocular complications. The best-corrected visual acuity was 1.0 preoperatively, on postoperative day 1, and at 3 months postoperatively; however, the patient's subjective visual disturbance, described as blurred and shimmering vision, improved after refixation. At 3 months postoperatively, the intraocular pressure was 17 mmHg, and the corneal endothelial cell density was 2826 cells/mm². This single case suggests that the Flanged Hook Technique may be a feasible option in selected anteriorly accessible cases of late IOL-capsular bag complex dislocation. However, this technique remains preliminary and off-label, and further cases with longer follow-up are required to evaluate its appropriate indications, durability, and long-term safety.

## Introduction

Late intraocular lens (IOL)-capsular bag complex dislocation is an important delayed complication after cataract surgery and can cause visual disturbance or progressive IOL instability [1,2]. It is often associated with zonular weakness related to pseudoexfoliation syndrome, high myopia, ocular trauma, uveitis, retinitis pigmentosa, prior vitreoretinal surgery, or capsular changes after posterior capsulotomy [1,2]. When clinically significant decentration, phacodonesis, vitreous prolapse, or progressive displacement develops, surgical intervention may be required to prevent further visual impairment or complete posterior dislocation.

Established surgical options include IOL explantation, pars plana vitrectomy-assisted management, and secondary IOL fixation by scleral suturing or flanged intrascleral fixation [1-4]. These procedures are effective and widely used, particularly when the IOL-capsular bag complex has completely dislocated posteriorly. However, IOL explantation can be technically demanding, and the method of removal depends on the IOL material and design; a large incision may be required in some cases [5].

Capsular bag-preserving approaches using capsular support devices, capsular anchors, capsular hooks, customized polypropylene hooks, and iris retractors have also been reported [6-17]. However, many of these methods require dedicated or modified devices, custom-made hooks, capsular tension segments, scleral suturing or scleral flaps, fibrin glue, posterior segment manipulation, or relatively complex intraocular maneuvers.

A clinical gap therefore remains for selected anteriorly accessible cases in which the existing IOL-capsular bag complex may still be preserved: whether such cases can be refixated using a simpler sutureless technique with relatively accessible instruments, without dedicated capsular fixation implants or complex scleral fixation maneuvers. This single-patient case report describes the Flanged Hook Technique, a sutureless anterior approach using an unmodified commercially available polypropylene iris hook for refixation of an anteriorly accessible late IOL-capsular bag complex dislocation.

## Case presentation

A 75-year-old man presented to our clinic with bilateral decreased vision and blurred vision. Slit-lamp examination showed bilateral cataracts. In the right eye, the uncorrected visual acuity was 0.2, improving to 0.7 with a manifest refraction of -2.00 diopters sphere/-0.50 diopters cylinder at 160 degrees. The axial length of the right eye was 25.91 mm, and the preoperative intraocular pressure (IOP) was 19 mmHg. The preoperative corneal endothelial cell density was 2659 cells/mm². There was no pseudoexfoliation, history of ocular trauma, or high myopia.

Bilateral cataract surgery was performed at our clinic. In the right eye, a Johnson & Johnson Vision PCB00V IOL (New Brunswick, NJ, US) was implanted in the capsular bag. The cataract surgery was uneventful, and no posterior capsule rupture, zonular dialysis, vitreous prolapse, or other intraoperative complication was observed. No early postoperative complication was noted.

At 3 months after cataract surgery, the uncorrected visual acuity in the right eye was 0.6, improving to 1.0 with a manifest refraction of -1.00 diopters sphere/-0.50 diopters cylinder at 160 degrees. Posterior capsule opacification subsequently developed, and neodymium: YAG laser posterior capsulotomy was performed approximately four months after cataract surgery.

Approximately one year after cataract surgery, vitreous prolapse into the anterior chamber was observed in the right eye. At that time, the IOL position remained satisfactory, and there was no marked IOL decentration or instability; therefore, the patient was observed.

**Figure 1:**
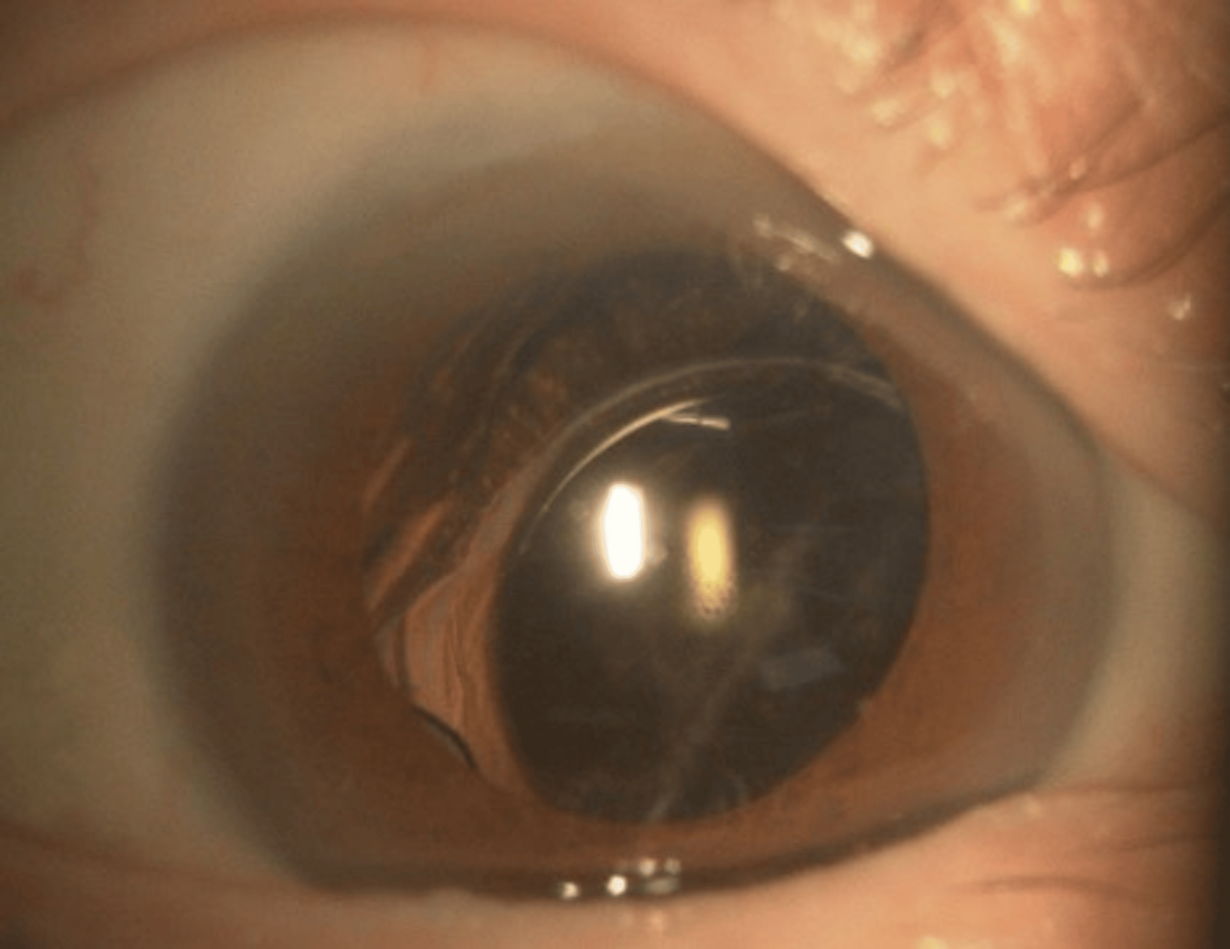
Preoperative slit-lamp photograph of the right eye The IOL-capsular bag complex was markedly decentered inferonasally without complete posterior dislocation into the vitreous cavity. Vitreous prolapse into the anterior chamber was also observed. The existing IOL was preserved within the capsular bag. IOL: intraocular lens

Approximately 2 years after cataract surgery, the uncorrected visual acuity in the right eye was 0.4, and the best-corrected visual acuity (BCVA) was 1.0 with a manifest refraction of -0.50 D. The IOP was 16 mmHg, and the corneal endothelial cell density was 2689 cells/mm². No Descemet's membrane folds were observed. Slit-lamp examination showed marked inferonasal decentration of the IOL-capsular bag complex with substantial phacodonesis. The IOL-capsular bag complex had not completely dropped into the vitreous cavity. Fundus examination showed no retinal tear, retinal detachment, or other clinically significant posterior segment abnormality (Figure 1).

Surgical repositioning and fixation were planned to prevent further dislocation. The patient described the subjective visual disturbance as blurred and shimmering vision. Although the IOL-capsular bag complex was decentered, it remained accessible from the anterior segment, and the anterior capsulorhexis margin was visible. These findings suggested that the existing IOL-capsular bag complex might be preserved, and a less invasive refixation approach without IOL explantation was therefore considered.

The patient was informed of standard options, including IOL explantation followed by secondary IOL fixation using scleral suturing or flanged intrascleral fixation. He was also informed that permanent use of a commercially available iris hook as a capsular fixation element was off-label and that its long-term safety had not been established. Written informed consent was obtained for surgery and for publication of the clinical information, images, and surgical video.

Preoperative mydriasis was achieved using tropicamide/phenylephrine ophthalmic solution (Mydrin-P; Santen Pharmaceutical Co., Osaka, Japan). The ocular surface was disinfected with povidone-iodine (Isodine 10% solution; Mundipharma K.K., Tokyo, Japan), and a sterile surgical eye drape was applied. The conjunctival sac was irrigated with iodine polyvinyl alcohol ophthalmic and eye-washing solution (PA-Iodo; Nitten Pharmaceutical Co., Nagoya, Japan). Local anesthesia consisted of topical 4% lidocaine (Xylocaine; Sandoz Pharma K.K., Tokyo, Japan) and a transconjunctival retrobulbar injection of 4 mL of 2% lidocaine. Intracameral anesthesia was not used.

Two side-port incisions were created at the 2 and 10 o'clock positions. A dispersive ophthalmic viscosurgical device (Viscoat; sodium hyaluronate and sodium chondroitin sulfate; Alcon Japan, Tokyo, Japan) was injected into the anterior chamber. A cohesive sodium hyaluronate ophthalmic viscosurgical device (hyaluronic acid Na ophthalmic viscosurgical device 1% 'Alcon'; Alcon Japan) was then injected between the IOL and the capsular bag to bluntly separate adhesions.

**Figure 2:**
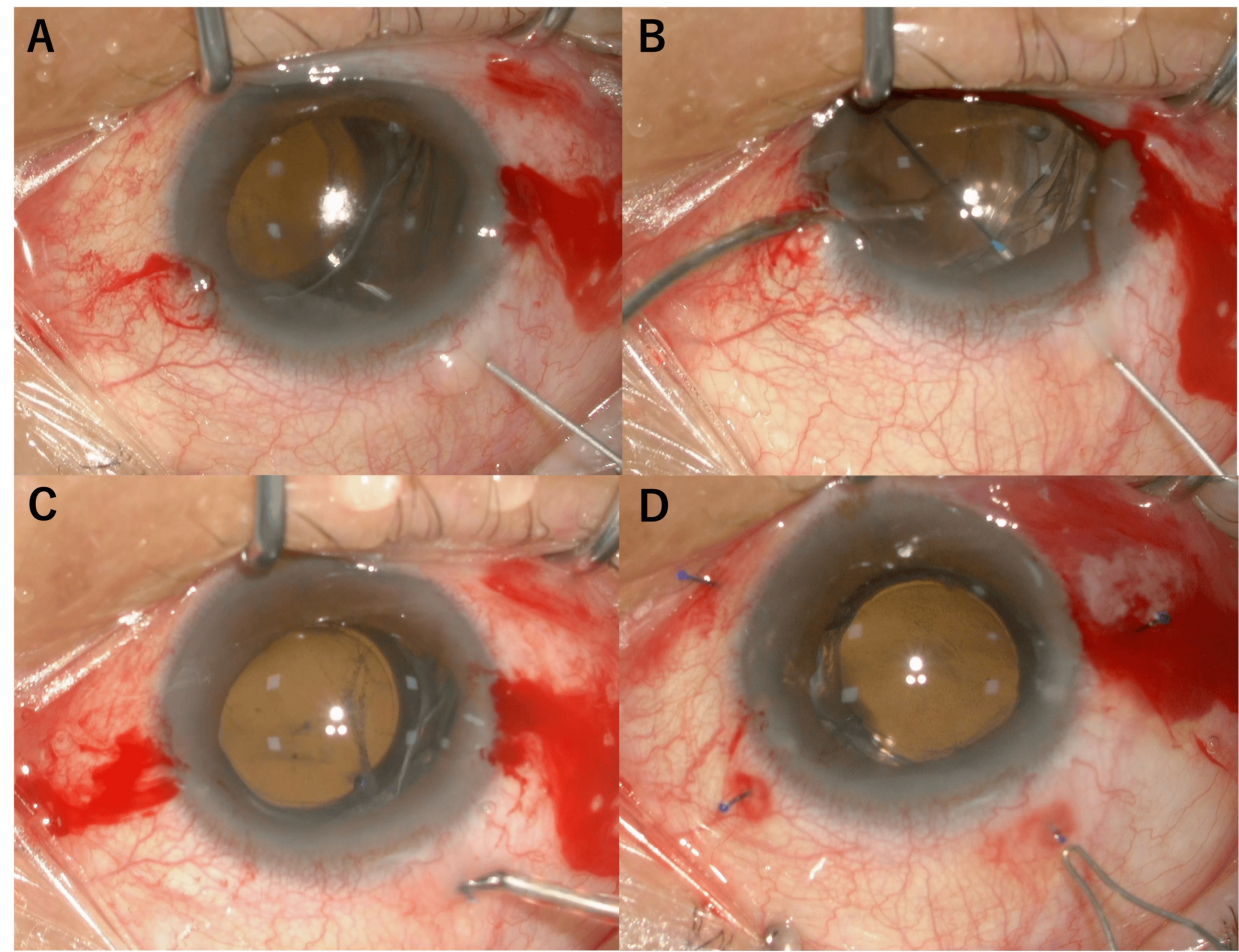
Intraoperative photographs showing the Flanged Hook Technique A: A 30-gauge thin-walled needle is inserted transconjunctivally 2 mm posterior to the limbus. B: The straight end of a polypropylene iris hook is inserted into the needle lumen. C: The hook is engaged with the anterior capsulorhexis margin and externalized by withdrawing the needle. (D) The externalized hook end is thermally flanged before intrascleral burial.

At the 4 o'clock position, a 30-gauge thin-walled needle (Tochigi Seiko Co., Ltd., Tochigi, Japan) was inserted transconjunctivally into the sclera 2 mm posterior to the corneal limbus. The needle was advanced into the anterior chamber in a relatively perpendicular direction to avoid capsular damage. A commercially available polypropylene Iris Hook Advance (Handaya Co., Ltd., Tokyo, Japan; length, 8.5 mm; diameter, 0.145 mm) was introduced through a side-port incision using capsulorhexis forceps, and the straight end of the iris hook was inserted into the lumen of the 30-gauge needle. The hook tip was engaged with the anterior capsulorhexis margin, and the needle was then withdrawn to externalize the outer end of the iris hook. The key intraoperative steps are shown in Figure 2.

The same maneuver was repeated at the 2-, 8-, and 10 o'clock positions. At each fixation point, a 30-gauge, thin-walled needle was inserted transconjunctivally through the sclera 2 mm posterior to the limbus, the straight end of the iris hook was introduced into the needle lumen, the hook was engaged with the anterior capsulorhexis margin, and the needle was withdrawn to externalize the hook. The four hooks were then used to recenter the IOL-capsular bag complex toward the pupillary center.

After the external end of each iris hook had been adjusted to an appropriate length, low-temperature cautery (Accu-Temp Cautery; Beaver-Visitec International Japan, Tokyo, Japan) was used to create a flange. Each flange was buried intrasclerally while the engagement of the hook with the capsulorhexis margin, the amount of capsular traction, and the centration of the IOL-capsular bag complex were carefully assessed.

Vitreous prolapse into the anterior chamber was removed using a 25-gauge vitrectomy cutter with the Constellation Vision System (Alcon Laboratories, Fort Worth, TX, USA). The ophthalmic viscosurgical device was aspirated, and the two side-port incisions were confirmed to be closed.

Because the IOL-capsular bag complex could be controlled from the anterior segment, full pars plana vitrectomy was not performed. The procedure was completed through two side-port incisions without a main corneal incision, IOL explantation, scleral suturing, scleral flap creation, fibrin glue, capsular tension ring implantation, capsular tension segment implantation, or a dedicated capsular fixation device. No apparent intraoperative capsular tear, iris injury, IOL damage, bleeding, or retinal complication occurred. The surgical procedure is demonstrated in Video 1.

**Video 1:** Surgical video of the Flanged Hook Technique A sutureless anterior-segment approach for late IOL-capsular bag complex dislocation is demonstrated. After creation of 2 corneal side-port incisions, OVD injection, and limited anterior vitrectomy, the straight end of a polypropylene iris hook was inserted into the lumen of a 30-gauge thin-walled needle placed transconjunctivally 2 mm posterior to the limbus. The hook was engaged with the anterior capsulorhexis margin, externalized by withdrawing the needle, thermally flanged with low-temperature cautery, and buried intrasclerally to achieve fixation of the IOL-capsular bag complex. IOL: intraocular lens; OVD: ophthalmic viscosurgical device

**Figure 3:**
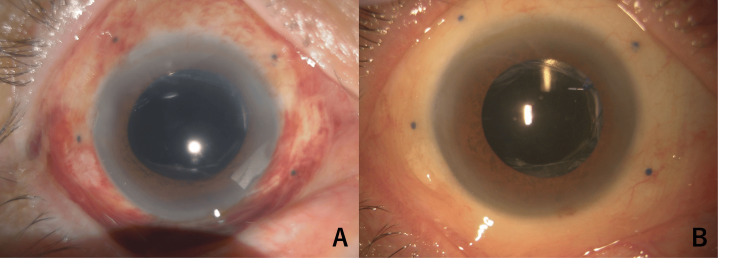
Postoperative slit-lamp photographs of the right eye A: On postoperative day 1, the IOL-capsular bag complex was well-centered, and the flanged hook remained engaged with the anterior capsulorhexis margin, without flange exposure or Descemet's membrane folds. B: At three months postoperatively, centration was maintained without flange exposure, conjunctival erosion, or recurrent decentration. IOL: intraocular lens

On postoperative day 1, the uncorrected visual acuity in the right eye was 0.7, and the BCVA was 1.0 with a manifest refraction of -0.75 -0.50 x 100. The IOP was 22 mmHg. Slit-lamp examination showed that the flanged hook remained engaged with the anterior capsulorhexis margin and that the IOL-capsular bag complex was well-centered without apparent IOL instability (Figure 3A). No flange exposure was observed, no Descemet's membrane folds were present, and anterior chamber inflammation was minimal. Fundus examination showed no significant abnormality.

At 3 months postoperatively, the uncorrected visual acuity was 0.9, and the BCVA was 1.0 with a manifest refraction of -0.75 D. The IOP was 17 mmHg. The cornea remained clear, and slit-lamp examination showed that the flanged hook remained engaged with the anterior capsulorhexis margin and that the IOL-capsular bag complex remained well-centered without apparent instability (Figure 3B). No flange exposure, conjunctival erosion, hook disengagement, capsular damage, or recurrent decentration was observed. The corneal endothelial cell density was 2826 cells/mm². Optical coherence tomography showed no cystoid macular edema, and fundus examination showed no retinal tear, retinal detachment, or other clinically significant posterior segment complication. The postoperative slit-lamp findings are shown in Figure 3. The patient reported improvement of the preoperative blurred and shimmering visual disturbance after refixation, although the BCVA was 1.0 preoperatively, on postoperative day 1, and at 3 months postoperatively. The key preoperative and postoperative clinical findings are summarized in Table 1. IOL-capsular bag centration, flange exposure, hook position, and anterior chamber inflammation were assessed clinically by slit-lamp examination. Phacodonesis and IOL-capsular bag instability were evaluated by slit-lamp examination and intraoperative observation. Posterior segment complications were assessed by dilated fundus examination and optical coherence tomography when appropriate. No standardized quantitative grading system or image-based measurement was used for IOL decentration, phacodonesis, or anterior chamber inflammation.

**Table 1:** Summary of preoperative and postoperative clinical findings IOL: intraocular lens

Clinical parameter	Before refixation surgery	Postoperative day 1	Postoperative month 3
Subjective symptoms	Blurred and shimmering visual disturbance	Improved or stable	Improved subjective visual disturbance
Uncorrected visual acuity	0.4	0.7	0.9
Best-corrected visual acuity (BCVA)	1.0 with -0.50 D	1.0 with -0.75 -0.50 x 100	1.0 with -0.75 D
Intraocular pressure	16 mmHg	22 mmHg	17 mmHg
IOL-capsular bag position	Inferonasal decentration with phacodonesis	Well centered	Well centered
IOL-capsular bag instability	Present	Absent	Absent
Anterior capsulorhexis margin	Visible and anteriorly accessible	Hook engaged	Hook remained engaged
Corneal endothelial cell density	2689 cells/mm²	Not assessed	2826 cells/mm²
Vitreous prolapse	Present in the anterior chamber	Not observed after anterior vitrectomy	Not observed
Anterior chamber inflammation	No significant inflammation	Minimal	No significant inflammation
Descemet's membrane folds	Not observed	Absent	Absent

## Discussion

This case demonstrates short-term anatomical stabilization of an anteriorly accessible late IOL-capsular bag complex dislocation using a sutureless anterior-segment approach. A commercially available polypropylene iris hook was engaged with the anterior capsulorhexis margin, externalized through a 30-gauge thin-walled needle, thermally flanged, and buried intrasclerally. The existing IOL-capsular bag complex was preserved, and centration was maintained without flange exposure or recurrent decentration during the limited three-month follow-up period. We refer to this procedure as the Flanged Hook Technique.

The present technique should be interpreted as a practical modification of previously reported capsular support and fixation concepts rather than as an entirely new category of surgery. Several methods have been described to stabilize or refixate the capsular bag or IOL-capsular bag complex, including capsular tension devices, capsular anchors, capsular hooks, and customized polypropylene fixation elements [6-13], as well as iris hook- or iris retractor-based scleral fixation approaches [14-17]. These reports support the general concept that the anterior capsulorhexis margin or capsular bag can be used as a fixation target. An experimental study also reported that polypropylene iris hooks can form flanges suitable for intrascleral fixation [17]. The specific contribution of the present case is the adaptation of an unmodified commercially available polypropylene iris hook as a flanged capsular fixation element in a late IOL-capsular bag complex dislocation that remained accessible from the anterior segment.

Compared with IOL explantation followed by secondary IOL fixation [1-5], this approach may reduce intraocular manipulation and avoid a large corneal or corneoscleral incision in carefully selected cases. It is not intended to replace pars plana vitrectomy or IOL explantation followed by secondary IOL fixation for complete posterior dislocation. Rather, it may serve as an intermediate anterior approach when the IOL-capsular bag complex remains salvageable, the anterior capsulorhexis margin can be visualized and engaged, and sufficient anterior vitreous management can be performed.

The postoperative Day 1 findings may support the minimally invasive nature of this approach, as the IOL-capsular bag complex was already well-centered, the BCVA was already 1.0, no Descemet's membrane folds were present, and anterior chamber inflammation was minimal. These early anatomical and functional findings may be related to the avoidance of IOL explantation and a large corneal incision. However, these findings should be interpreted cautiously because they are derived from a single patient and short-term follow-up only. The absence of early complications in this case does not establish the long-term safety or durability of the technique.

The technique has important prerequisites and limitations. The IOL-capsular bag complex must not have completely dislocated into the vitreous cavity, and the anterior capsulorhexis margin must remain visible and accessible from the anterior segment. Excessive traction on the anterior capsulorhexis margin may cause capsular tearing, hook disengagement, or recurrent decentration. Therefore, careful adjustment of the amount and direction of traction is essential.

Postoperative risks related to the flange and the permanent intraocular use of an iris hook also require attention. Direct ocular compression or eye rubbing may theoretically push the hook intraocularly or disturb its engagement with the capsulorhexis margin, particularly in the early postoperative period. Long-term follow-up is also required to monitor for flange exposure, conjunctival erosion, pigment dispersion, IOP elevation, chronic inflammation, hook dislocation, and recurrent IOL-capsular bag complex decentration. Because the permanent use of a commercially available iris hook as a capsular fixation element is off-label, long-term interaction among the hook, capsular bag, IOL, iris, and angle structures remains uncertain.

This report has several limitations. It describes a single case with only three months of follow-up, and no standardized quantitative grading system or image-based measurement was used for IOL decentration, phacodonesis, or anterior chamber inflammation. Further cases with longer follow-up are required to clarify the appropriate indications, reproducibility, durability, and long-term safety of this preliminary technique.

## Conclusions

This single case suggests that the Flanged Hook Technique using a commercially available polypropylene iris hook may be a feasible option for sutureless refixation of selected anteriorly accessible late IOL-capsular bag complex dislocations. In this case, centration was maintained without flange exposure during the limited three-month follow-up period. However, this technique remains a preliminary and off-label approach, and longer follow-up with additional cases is required to clarify its appropriate indications, durability, and long-term safety.
